# Zanamivir exposure in healthy rats and rats with acute lung injury

**DOI:** 10.1080/07853890.2025.2534523

**Published:** 2025-07-20

**Authors:** Anežka Klouček, Jaroslava Šmardová, Michaela Sklenárová, Alexandra Dvořáková, Karel Chalupský, Pavel Ryšánek, Martina Nora Odložilíková, Aleš Bartůněk, Vít Perlík, Helena Mertlíková-Kaiserová, Pavel Michálek, Martin Šíma, Ondřej Slanař

**Affiliations:** ^a^ Institute of Pharmacology, First Faculty of Medicine, Charles University and General University Hospital in Prague, Prague, Czech Republic; ^b^ Institute of Organic Chemistry and Biochemistry, Academy of Sciences of the Czech Republic, Prague, Czech Republic; ^c^ Synavia S.R.O, Prague, Czech Republic; ^d^ Department of Anaesthesiology, Resuscitation and Intensive Care Medicine, General University Hospital in Prague, Prague, Czech Republic

**Keywords:** Bronchoalveolar lavage, bioavailability, inhalation drug delivery, lung inflammation, respiratory diseases

## Abstract

**Objectives:**

The aim of this study was to evaluate the pharmacokinetics and lung penetration of zanamivir in healthy rats and rats with lipopolysaccharide (LPS)-induced acute lung injury (ALI).

**Materials and methods:**

Three pharmacokinetic (PK) studies have been conducted to evaluate systemic PK and local exposure of zanamivir in male Wistar rats (*n* = 62, 16 weeks old). Zanamivir was administered to healthy rats and rats with LPS-induced ALI intravenously (IV) and by inhalation (INH) *via* nebulisation. Serum and bronchoalveolar lavage (BAL) fluid concentrations were analysed to assess drug permeation across barriers. All zanamivir concentrations were determined using the HPLC–MS/MS method.

**Results:**

The concentrations of zanamivir in BAL after IV dosing were approximately 3.1-, 4.0- and 5.0-fold higher in healthy animals compared with ALI at 30, 60 and 240 min after dosing, respectively (*p* = 0.005, 0.001 and 0.016). Zanamivir permeation between BAL fluid and serum was compared for IV and INH administrations, revealing that the BAL AUC_30–240_ following IV administration was 6.5-fold lower than after INH. Furthermore, the AUC_30–240_ in BAL fluid after IV administration was approximately 3.3 times higher in healthy animals than those with ALI (35,815 vs. 10,886 ng/mL × h). ALI also reduced the rate and extent of systemic absorption compared to healthy conditions. The absolute bioavailability of nebulised zanamivir was 1.91%.

**Conclusions:**

Our findings confirm PK superiority of INH administration to achieve local intrapulmonary exposition and indicate that ALI significantly impairs zanamivir penetration into the lungs from systemic circulation.

## Introduction

Influenza is a common acute respiratory infection worldwide, with an estimated 3–5 million severe cases per year. It remains a major global public health concern, causing up to 650,000 respiratory deaths each year. Hospitalisation and death are more prevalent among high-risk groups, including the elderly, young children and individuals with underlying health conditions. Influenza viruses spread easily through infectious droplets, and seasonal epidemics place a significant burden on healthcare systems. To mitigate these consequences, neuraminidase inhibitors are used as the key drugs for the influenza prophylaxis and treatment [1–3].

Zanamivir is the first neuraminidase inhibitor available for the treatment of influenza infections. It targets neuraminidase enzyme in both influenza A and B viruses. By binding to and inhibiting this enzyme, zanamivir prevents the virus from cleaving sialic acid residues on the host cell surface, which is necessary to release and spread newly formed progeny viruses. Neuraminidase inhibition thus limits the release of newly formed viruses from the host cell surface and reduces the ability of influenza viruses to spread within the respiratory tract. Subsequently, the duration and severity of the disease are reduced [4–6].

Due to this mechanism of action and the primary site of influenza virus replication within the respiratory tract epithelium, achieving sufficient local drug concentrations at the site of infection is critical for drug efficacy [7,8].

Currently, there are two approved zanamivir-containing drug formulations for the treatment of influenza; Dectova^®^ may be administered *via* IV infusion, and Relenza^®^ is approved for INH delivery using a Diskhaler DPI device. Due to the poor oral bioavailability, oral zanamivir is not available.

The treatment is generally recommended as early as possible for patients with confirmed or suspected influenza who have severe, complicated, or progressive disease or are at high risk of influenza complications. During the recent influenza seasons, 9 out of 10 people hospitalised with influenza had at least one underlying health condition, while chronic respiratory illness, neuro-muscular diseases or obesity belong among the most frequent ones [9]. These comorbidities in many patients make the use of approved zanamivir DPI Diskhaler device difficult as it is classified as a low-resistance device requiring a relatively high peak inspiratory flow rate of >60 L/min to sufficiently aerosolise the drug for the efficient drug delivery [10]. This high respiratory effort often cannot be achieved by many patients with pulmonary disease or the elderly [11,12]. Therefore, the systemic administration of zanamivir *via* IV infusion is frequently preferred.

This administration relies on the ability of zanamivir to penetrate biological barriers, such as the alveolar–capillary interface, to reach the site of infection in the lungs. Lung permeability is, however, determined by many factors, such as drug physicochemical properties [13,14] and the conditions within the respiratory tract, which, when altered due to the disease, may affect the drug availability at the site of action [15].

Our understanding of drug permeability between the central compartment (blood) and lung tissues is incomplete, especially in patients suffering from pulmonary diseases. Lower intrapulmonary concentration of orally administered levofloxacin was observed in patients with pulmonary fibrosis, while the concentrations reached effective drug levels against common respiratory pathogens [16]. Highly variable lung penetration of meropenem has been observed in patients with ventilator-associated pneumonia [17], and inadequate exposure to ciprofloxacin was observed in patients with COPD when treated with a recommended dose [18].

The lung penetration of zanamivir in respiratory diseases remains unknown as the few relevant pharmacokinetic data are reported only in healthy volunteers [5]. Both approved dosage regimens for zanamivir, i.e. 600 mg IV q12h and DPI 10 mg q12h, resulted in comparable epithelial lining fluid concentrations in healthy subjects of 2.7 and 1.3 µM, respectively. However, there is no data on pulmonary zanamivir exposure in patients with influenza after IV drug dosing.

Our hypothesis was that zanamivir pharmacokinetics (PK) can be affected by lung physiology altered due to the inflammation, leading to an impaired drug permeability across the barriers. Thus, we expected that local lung exposure to zanamivir might be modified in subjects suffering from acute respiratory diseases when the drug was administered *via* IV infusion or another systemic route. The aim of the study was to evaluate the PK of zanamivir in a rat model under both healthy conditions and lipopolysaccharide (LPS)-induced acute lung injury (ALI). By comparing drug concentrations in the BAL fluid and serum after both inhalation and IV drug administration, we aimed to understand how ALI affects zanamivir distribution across lung barriers in healthy and ALI animals.

## Materials and methods

### Experimental design

A series of PK studies were performed in accordance with FDA and EMA guidelines [19,20]:

- The absolute bioavailability was evaluated in a randomised, laboratory-blinded, single-dose, two-period, fixed-sequence PK study. The rats (*n* = 6) were dosed IV zanamivir (5.1 mg/kg of body weight) via the left jugular vein in the first period. Blood samples (120 µL) were taken 2, 10, 20, 30, 60, 120, 240 and 480 min after dosing through the right jugular vein catheter. After a 48-hour wash-out period between the first and second periods, zanamivir INH (5.1 mg/kg of body weight) was conducted as described below. Blood samples were collected at the end of nebulisation and at 10, 20, 30, 60, 120, 240 and 480 min after dosing via the right jugular vein catheter. After each sampling, the volume of withdrawn blood was replaced by an equal volume of saline and 50 µL of heparinised saline flush (1000 IU/mL). Blood samples were centrifuged at 2000 g, 4 °C, for 10 min. The separated serum was stored at –20 °C until further analysis.
- The permeation of zanamivir between lung lining fluid and systemic circulation was determined from parallel BAL and blood samples. Zanamivir (5.1 mg/kg of body weight) was administered by INH or IV to healthy rats (*n* = 12; 4 rats for each time point) and rats with ALI (*n* = 12; 4 rats for each time point). Drug administration procedures, and induction of ALI and BAL samplings were performed as described below. Blood and BAL samples were taken at 2, 60, and 240 min after the end of drug nebulisation or at 30, 60, and 240 min after IV bolus. Blood samples were centrifuged at 2000*g*, 4 °C, for 10 min. The separated serum was stored at –20 °C until further analysis.
- To compare systemic exposure between healthy and ALI rats (*n* = 8), a two-period, fixed-sequence PK study of nebulised zanamivir was performed with right jugular cannulation. In both periods, zanamivir (5.1 mg/kg of body weight) was administered by INH as described below. Period 1 was conducted in healthy animals, and subsequently, ALI was induced by LPS, as described below. Period 2 started 24 hours after ALI induction. Blood samples (120 µl) were collected at the end of nebulisation and at 10, 20, 30, 60, 120, 240, and 480 min after the end of drug administration in both periods. After each sampling, the volume of withdrawn blood was replaced by an equal volume of saline and 50 µL of heparinised saline flush (1000 IU/mL). Blood samples were centrifuged at 2000 g, 4 °C, for 10 min. The separated serum was stored at –20 °C until further analysis.

In all experiments, the dose of zanamivir was 5.1 mg/kg of body weight according to the traditional corresponding surface area–dosage conversion based on the literature [21,22].

All rats were euthanised using T61 at the end of each experiment.

### Experimental procedures

#### Nose-only inhalation exposure

A nose-only exposure system (inExpose, SCIREQ Scientific Respiratory Equipment Inc., Canada) was used for zanamivir aerosol inhalation. The duration of INH was until the entire drug solution (2 mL) was nebulised, typically lasting around 10 min. Each rat was immobilised in a restrainer connected to the exposure tower. This setup ensured that only the nose and mouth were exposed to the aerosol with only minimum skin or fur drug contamination. Zanamivir solution was nebulised with an Aeroneb nebuliser and evenly distributed to each exposure chamber through the exposure tower for rats to inhale.

#### Implantation of jugular catheters

Animals underwent surgery under general anaesthesia three days before the experiment. During surgery, one or both external jugular veins were cannulated with polyurethane catheters (3 Fr, Instech laboratories, PA, USA). One catheter was used for repeated blood sampling, and the other allowed IV administration of the drug. Heparin was used as a perioperative anticoagulant, and carbethopendecinium bromide was used as an antiseptic treatment. Subcutaneous ketoprofen (5 mg/kg) was used as a perioperative analgesic. After the surgical cannulation, the rats were kept under a heating lamp and then placed in individual cages.

#### LPS lung injury induction

The non-surgical intratracheal instillation of LPS was performed in order to induce ALI as described previously [23]. In brief, the tongue of each anaesthetised rat was gently extended to one side with forceps while the rat was positioned on a platform with its incisors attached to a string. The trachea was visualised, and 500 µL of LPS dissolved in saline (5 mg/kg) was administered slowly into the trachea using a pipette. The rats were observed after LPS administration to ensure a complete recovery from anaesthesia.

#### BAL collection and cytology analysis

BAL fluid sampling was performed in euthanised animals according to the protocol described by Kim et al. [24]. Briefly, the trachea was exposed and an incision between two cartilage rings was performed. A polyethylene catheter (inner diameter of 1.73 mm, outer diameter of 2.17 mm; Terumo corporation, Japan) connected to a syringe containing 2 mL of 0.09% NaCl at 37 °C was inserted into the trachea. BAL fluid samples were obtained by infusing NaCl and then aspirating the maximum volume. The flush was repeated two times. The collected samples were centrifuged at 1000*g* for 6 min. Aliquots of the supernatant from the first BAL were collected and stored at −20 °C until further analysis. Cell pellets were resuspended for total count and cell differentials. Total cell numbers were counted with a cell counter (LUNA, Logos Biosystems Inc., Korea). Differential cell counting was performed after spinning the cells collected from both BALs in a cytocentrifuge (Centric250, Domel, Slovenia) and May–Grunwald–Giemsa staining.

#### Animals and ethics statement

All animal experiments were in accordance with ARRIVE guidelines and approved by the animal Ethics Committee of the by the First Faculty of Medicine, Charles University and by the Ministry of Education, Youth, and Sports, Czech Republic under MSMT-26321/2023-5.

Male Wistar rats (*n* = 62, 16 weeks old) purchased from Velaz (Czech Republic) were housed under controlled conditions of temperature (22 ± 2 °C) and humidity (50 ± 10%) with a 12-h light–dark cycle. All animals were given free access to water and a standard chow diet.

#### Reagents

Isoflurane (IsoFlo 100% inh. vap. Liq., Zoetis, Czech Republic), ketamine (Narkamon 100 mg/mL inj. sol., Bioveta a.s., Czech Republic) and xylazine (Rometar 20 mg/mL, inj. Sol.; Bioveta a.s., Czech Republic) were used to anaesthetize the animals. Heparin (Heparin Léčiva 5000 IU/mL inj. sol., Zentiva, Czech Republic), carbethopendecinium bromide (Ophthalmo-Septonex eye ointment, Zentiva, Czech Republic) and ketoprofen (Ketodolor 100 mg/mL inj. sol., LeVet Pharma b.v., the Netherlands) were used as a part of perioperative care. Zanamivir (Dectova 10 mg/mL, GlaxoSmithKline Trading Services Limited, Ireland) was used for all experiments. LPS from *Escherichia coli* (055:B5, Sigma-Aldrich, MO, USA) were purchased from Sigma (MO, USA). T61 (inj. sol., Intervet International b.v., the Netherlands) was used for animal euthanasia.

### Bioanalytical assays

#### Zanamivir analysis in serum and BAL

All stock and working solutions of zanamivir were prepared in Milli-Q water. Calibration standards and quality control samples were prepared by spiking 20 µl of blank rat serum (for serum samples) or saline (for BAL samples) with 2.5 µl of the appropriate zanamivir working solution to obtain concentrations of zanamivir in the unprocessed sample from 5 to 1000 ng/ml (7 non-zero point-calibration). QC samples were prepared at three zanamivir levels – low (15 ng/ml), medium (250 ng/ml) and high (750 ng/ml). Accordingly, 20 µl of experimental serum and BAL samples were spiked with 2.5 µl Milli-Q water.

All samples (calibration, QC, experimental) were then extracted with three volumes of ice-cold methanol (67.5 µl) containing 40.63 ng/ml of stable isotope-labelled zanamivir (SIL–zanamivir) as an internal standard. The samples were first vortexed for 1 min at room temperature and then centrifuged at 2,000×*g* at 8 °C for 20 min. The supernatants were analysed by LC–MS/MS.

Briefly, a 2 µL aliquot of the extracts was injected into the chromatographic system Agilent 1260 Infinity II. LC separation was performed using an iHILIC-(P) Classic 5 µm 50 × 2.1 mm column protected by iHILIC-(P) Classic 5 µm 20 × 2.1 mm guard column (Hilicon). A gradient elution was employed, with mobile phase A consisting of 10 mM ammonium formate in Milli-Q water (pH 6.0) and mobile phase B comprising 90% acetonitrile The gradient employed was as follows: 0–3 min linear decrease of solvent B from 100% to 44%, 3–6 min isocratic hold on 44% B, 6–7 min linear increase from 44% to 90% B and 7–11 min reset and equilibration at 100% B. The flow rate was set to 0.3 ml/min. MS/MS analysis was performed on Triple Quad^TM^ 7500 (Sciex) utilizing multiple reactions monitoring 333.06 to >60.2 for zanamivir and 336.08–>63.0 for SIL–zanamivir. The mass–chromatograms reconstructed from the ion profiles of zanamivir (m/z), SIL–zanamivir (m/z) were integrated and the area ratios of zanamivir/SIL–zanamivir were used to construct calibration curve and to back-calculate the concentration of zanamivir in QC samples and experimental (serum and BAL) samples. Weighted linear regression method was employed. Samples below LLOQ (5 ng/ml) were considered zero. Samples above ULOQ (1000 ng/ml) were 10x diluted and re-analysed.

#### Urea analysis in serum and BAL

Urea concentrations were measured in serum and BAL samples using Blood Urea Nitrogen (BUN) Colorimetric Detection Kit (EIABUN, intra-assay precision CV = 1.9–2.8%, inVitrogen, ThermoFisher, MA, USA).

Detection of inflammatory markers in BAL**:** The levels of TNF-α (ab236712, intra-assay precision CV = 3.9%, Abcam, Cambridge, UK) and CXCL-1 (ab219044, intra-assay precision CV = 10.1%, Abcam, Cambridge, UK) from BAL samples were determined using an enzyme-linked immunosorbent assay (ELISA) kits according to the manufacturer’s instructions. The optical density value was detected using the continuous wavelength multifunctional microplate reader Tecan Sunrise^TM^ (Tecan Austria GmbH, Austria).

#### Data analysis and statistics

The sample size was chosen based on the FDA guideline for bioequivalence studies: 6 animals per group for cross-over study design for descriptive study (the absolute bioavailability study) and 12 animals for the parallel group design (the permeation study) [19]. The sample size for comparing pre- and post-ALI exposure was estimated based on an expected 30% difference between conditions, with 80% power and alpha of 0.05, resulting in a required sample size of 8 animals.

PK analysis was determined by a non-compartmental model using Phoenix WinNonlin^®^ (Certara, NJ, USA). The area under the curve (AUC_last_) from time 0 to the last sampling time was calculated using the linear–trapezoidal method. The maximum serum concentration (*C*_max_) and the corresponding time (*T*_max_) were directly observed. For statistical analysis, *C*_max_ and AUC_last_ were log-transformed. Both parameters and absolute bioavailability (*F*) are reported as geometric means ± 90% confidence interval (CI). *T*_max_ is presented as a median ± interquartile range (IQR), and other PK parameters are represented as mean ± standard deviation (SD). The mean values of PK profiles ± SD were used for graph plotting.

AUC_last_ and *C*_max_ values were compared using a paired *t* test with a 90% CI for the ratio of geometric means. The model included fixed effects of treatment. Median *T*_max_ values were compared using the Wilcoxon matched-pairs signed rank test, while mean concentrations at each sampling time were analysed using a paired *t* test.

Zanamivir concentrations measured in BAL samples were adjusted by a dilution factor obtained from urea measurement in serum and BAL according to *Rennard* et al. by the following formula [25]: *c*_BAL_ = *c*_BALobserved_× (Urea_serum_/Urea_BAL_), where *c*_BAL_ represents the calculated concentration of zanamivir in BAL fluid; *c*_BALobserved_ refers to the zanamivir concentration measured in BAL sample; Urea_BAL_ and Urea_serum_ correspond to the urea concentrations determined in BAL sample and serum, respectively.

The concentration ratios were compared using an unpaired *t* test for each sampling time. The BAL/serum concentration and serum/BAL concentration ratios were used to describe drug permeability between lung lining fluid and blood after IV and INH administration, respectively.

The levels of inflammatory markers (TNF-α and CXCL-1), the total number of cells, and differential cell counts in BAL fluid were analysed using an unpaired *t* test to compare differences between the healthy and ALI groups. Descriptive statistics (mean ± SD) was used to summarise the data.

A paired *t* test was used to evaluate the difference between pre- and post-ALI induction weights within the same group for body weight comparisons.

All comparisons were performed in GraphPad Prism version 9.1.0 (GraphPad Software, CA, USA), and statistical significance was considered at *p* ≤ 0.05. No animals have been excluded from analyses.

## Results

### The absolute bioavailability study

**Figure 1.**
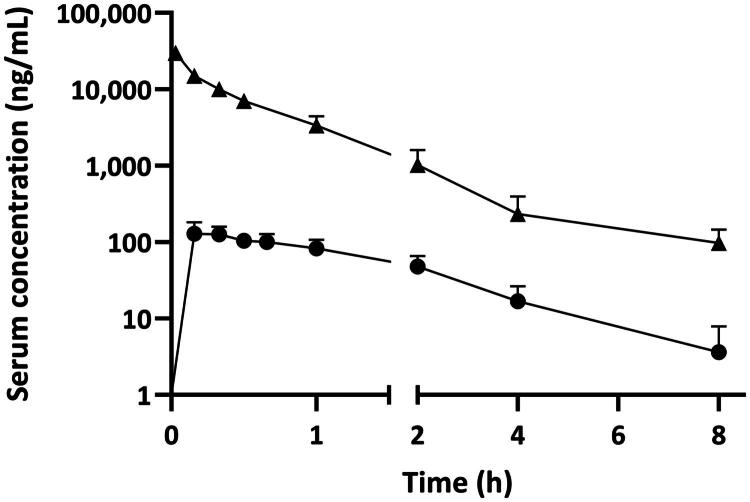
Concentration–time profiles of zanamivir after intravenous (triangles) and inhalation (circles) administration in healthy rats on a semilogarithmic scale. Symbols represent mean ± SD concentrations.

The mean serum concentration profiles of zanamivir in rats after IV and INH administration are presented in Figure 1. The PK parameters of zanamivir following IV and INH administrations are shown in Table 1. The absolute bioavailability of zanamivir after inhalation was determined to be 1.91%.

**Table 1.** Pharmacokinetic parameters of zanamivir following intravenous (IV) and inhalation (INH) administration in healthy rats.

Route	IV	INH
AUC_last_ (ng/mL × h)	13,275.1 (11 360–15 513)	273.9 (213.7–351.0)
*C*_max_ (ng/mL)	NA	149.2 (112.3–198.2)
*T*_max_ (min)	NA	20 (13–21)
*F* (%)	NA	1.91 (1.41–2.59)
λz (1/min)	0.0093 ± 0.0025	0.0078 ± 0.0026
*T*_1/2_ (min)	79.7 ± 23.7	97.6 ± 29.9
Vss (mL)	354.1 ± 114.9	NA
Cl (mL/min)	3.11 ± 0.63	NA

The area under the curve (AUC_last_), maximum concentration (*C*_max_), and absolute bioavailability (*F*) are presented as geometric mean values with 90% confidence intervals. Time to maximum concentration (*T*_max_) is presented as a median ± interquartile range. Elimination rate constant (λz), half life (*T*_1/2_), volume of distribution (Vss), and clearance (Cl) are presented as mean values ± SD.

### Penetration study of zanamivir into the lung lining fluid

**Figure 2.**
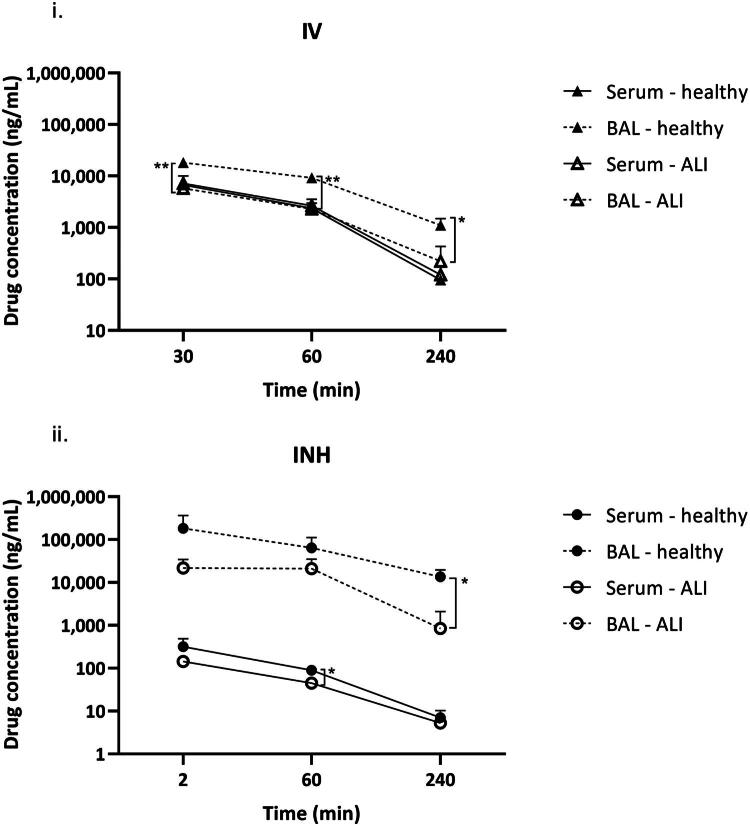
Zanamivir serum concentrations (solid line) and concentrations in bronchoalveolar lavage (dashed line) after intravenous (IV; triangles) and inhalation (INH; circles) administration in healthy rats (closed symbols) and rats with induced acute-lung injury (ALI) (open symbols) over time after the end of drug administration on a semilogarithmic scale. Symbols represent mean ± SD concentrations; *(*p* ≤ 0.05); **(*p* ≤ 0.01).

The zanamivir serum concentrations after IV administrations were not significantly different between the healthy and ALI groups. The concentrations of zanamivir in BAL after IV dosing were approximately 3.1-, 4.0- and 5.0-fold higher in healthy animals compared with ALI at 30, 60 and 240 min after dosing, respectively (*p* = 0.005, 0.001 and 0.016) (Figure 2).

After INH, the mean serum concentrations of zanamivir were approximately 2.2-, 2.0- and 1.3-fold higher in healthy animals compared with ALI at 2, 60 and 240 min after the dosing, respectively. Statistical significance has been reached only for the 60-min interval (Figure 2). Mean BAL concentrations of zanamivir were approximately 8.4-, 3.1- and 16.0-fold higher in healthy animals compared with ALI at 2, 60 and 240 min after inhalation dosing, respectively. Statistical significance has been reached only for the 240-min interval.

**Figure 3.**
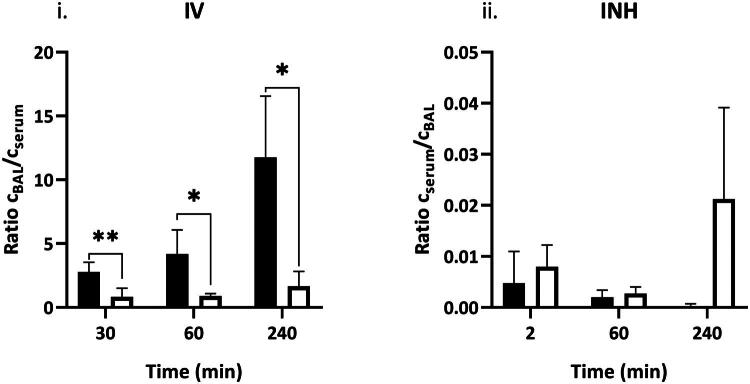
Zanamivir BAL:serum and serum:BAL concentration ratios after intravenous (IV) resp. inhalation (INH) administration in healthy rats (solid bars) and rats with induced acute-lung injury (open bars) over time after the end of drug administration. The values are represented as mean ± SD. BAL (bronchoalveolar lavage). *(*p* ≤ 0.05), **(*p* ≤ 0.01).

The ratio of zanamivir concentration in BAL:serum was significantly higher at all time points in the healthy group compared to the ALI group after IV administration. The ratio increased 4-fold between 30 and 240 min post dose in healthy animals, while the increase was only 2-fold in the ALI group (Figure 3i). After INH, the ratios of zanamivir serum:BAL concentrations were not significantly different between healthy rats and rats with induced ALI (Figure 3).

### Systemic exposure after inhalation administration of zanamivir – effect of ALI induction

**Figure 4.**
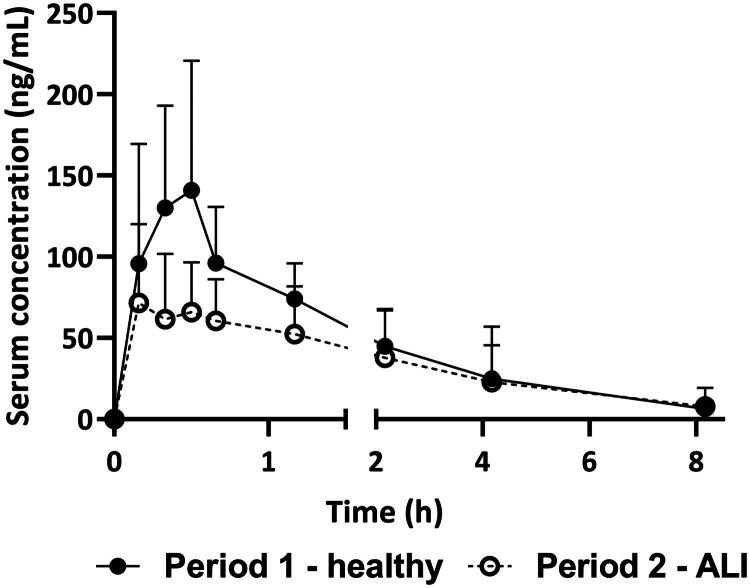
Concentration–time profiles of inhaled zanamivir in rats before (period 1 – healthy) and after induction of ALI (period 2 – ALI). Symbols represent mean ± SD concentrations.

PK parameters AUC_last_, *C*_max_ and *T*_max_ of inhaled zanamivir in healthy rats and the corresponding values in the same animals after ALI induction are summarised in Table 2. The systemic exposure to zanamivir was significantly lower after the ALI induction compared to the healthy state. The mean ± SD AUC_0–30_ describing exposure to gastric emptying time was significantly higher in healthy group 43.9 ± 22.1 compared with ALI 26.1 ± 15.0 (*p* = 0.01). The mean serum concentration profiles of zanamivir in healthy and ALI states are presented in Figure 4.

**Table 2.** Pharmacokinetic parameters of zanamivir before (healthy state; period 1) and after the induction of ALI (period 2).

Period	AUC_last_ (ng/mL × h)	*C*_max_ (ng/mL)	*T*_max_ (min)	Slope_abs_
1 (Healthy)	273.0 (196.6–379.3)	142.3 (102.6–197.3)	27 (14–34)	5.7 (5.3–6.1)
2 (ALI)	208.7 (146.1–298.2)**	74.6 (53.4–104.2)**	16 (12–31)	4.9 (4.4–5.3)**

The area under the curve (AUC_last_), maximum concentration (C_max_) and slope of linear regression of semi-ln concentration–time points during absorption phase (Slope_abs_) are presented as geometric mean values with 90% confidence intervals. Time to maximum concentration (T_max_) is presented as a median ± interquartile range. ** (*p* ≤ 0.01).

The rats’ body weight was evaluated before and 24 h after LPS induction as one of the parameters of ongoing ALI. The average body weight loss ± SD in all groups (PK study, penetration studies) was 6 ± 2% (Supplementary Figure 1).

Inflammatory marker levels from BAL samples were compared between healthy rats and rats after LPS induction. The levels of TNF-α and CXCL-1 in groups with ALI were significantly higher than those in healthy groups (Supplementary Figure 2).

The mean total number of cells obtained from the BAL sample was 1.52 × 10^6^ ± 9.44 × 10^5^cells/mL obtained from healthy animals and 6.02 × 10^6^ ± 6.44 × 10^6^cells/mL from rats with induced ALI (p = 0.016). Alveolar macrophage was the predominant cell type identified in healthy animals (Supplementary Figure 3 left), with an 83% representation, while in rats with ALI, there were only 18% of macrophages. On the other hand, neutrophils (80%) were the predominant cell type in rats with induced ALI (Supplementary Figure 3 right) as a manifestation of inflammation. BAL from healthy animals contained 6% of neutrophils. The remaining cells were identified as lymphocytes, with 11% and 2% in healthy rats and rats with ALI, respectively.

## Discussion

The LPS-induced ALI animal model is a widely used model of lung inflammation. The model involves lung injury induced by intratracheal instillation of LPS, which triggers an intrapulmonary inflammatory response involving leukocyte mobilisation, release of proinflammatory cytokines, and consequent lung injury [26]. The increased levels of inflammatory markers (TNF-α and CXCL-1), increased cellularity of the BAL, and the predominance of neutrophils in BAL were observed in our results and confirm the induction of a strong inflammatory response in the ALI groups. These results confirm lung inflammation and damage in line with previous reports. Ulich et al. demonstrated that LPS induced TNF-α expression and caused the accumulation of neutrophils in BAL fluid [27]. In studies conducted by Chuang et al. [28] and Davino-Chiovatto et al. [29], the enhanced levels of inflammatory markers (including TNF-α and CXCL-1), the influx of neutrophils and the pulmonary oedema were reported in male rats using similar LPS-induced ALI protocols. During LPS-induced ALI, the lung tissue is affected by pathophysiological processes similar to those seen in pneumonia, i.e. oedema, changes in blood perfusion or inflammatory exudate in the intra-alveolar area [23,30,31]. However, following intratracheal LPS instillation, the systemic inflammatory response associated with infectious pneumonia is not present [32]. Since our aim was to investigate the importance of local inflammatory changes on pulmonary drug permeation, the absence of systemic inflammation does not diminish its relevance. Rather, it offers the advantage of isolating lung-specific effects without confounding influences from systemic responses.

The permeation of drugs across the pulmonary barrier is a poorly understood factor that may be important for the treatment of pulmonary processes. There are few studies that described permeation between the systemic compartment and BAL for tigecycline, gentamicin, and tobramycin [33,34]. The lung penetration of subcutaneously administered tigecycline [33] or IV administered gentamicin and tobramycin [34] was enhanced in animals with model lung inflammation.

Contrary to these observations, our results show that the permeability of zanamivir between lung lining fluid and systemic circulation in either direction was decreased in animals with model lung inflammation. These conclusions are drawn from the ratios of zanamivir BAL:serum concentrations, where the healthy subjects had values indicating higher drug penetration from the blood to the lungs compared with subjects with ALI. The AUC_30–240_ in BAL fluid was compared between IV and INH administrations, revealing that the AUC following IV administration was 6.5-fold lower than after INH. Furthermore, the AUC_30–240_ in BAL fluid after IV administration was approximately 3.3 times higher in healthy animals than those with ALI (35,815 vs. 10,886 ng/mL × h Further confirmation has been generated by the systemic exposure study conducted after INH drug delivery in a fixed sequence design implementing drug exposure evaluation in the same subjects in a healthy state and after the induction of ALI. The mean ± SD of C_max_ during ALI conditions was 53.1 ± 9.9%, and the mean ± SD AUC_last_ was 77.6 ± 13.7% compared to the healthy state, indicating a significant alteration in the PK profile due to the pathophysiological state. Using this study design, we excluded inter-subject variability, and confirmed the observations of decreased zanamivir lung permeability in ALI subjects from the permeation study, which was performed using sparse sampling in parallel study groups.

The divergence between our observations and results by Cardon et al. and Valcke et al. [33,34] may be caused by the fact that physicochemical properties including polarity, solubility as well as permeability limit the distribution of zanamivir belonging to BCS class IV, while tigecycline, gentamicin, and tobramycin all belong to BCS class III compounds, i.e. highly soluble, low permeable drugs. In the inflammatory altered tissues characterised not only by higher perfusion but also by oedema and high exudation, substances with different solubilities may be distributed differently between compartments.

After INH in the permeation study, the ratios of serum to BAL concentrations were <1 (Figure 3ii), which means only a small fraction of the drug gets to the systemic circulation. The ratios of serum:BAL concentrations in rats with induced ALI trended higher compared to healthy animals after INH, although it did not reach statistical significance. We assume that this result is due to the faster absorption from the site of inhalation in healthy animals, where a rapid decrease in serum concentrations was observed in the later time points due to relatively fast systemic elimination. Conversely, in animals with ALI, the absorption was delayed, which was consistent with the slope of linear regression of semi-ln concentration–time points during absorption phase of 5.7 vs 4.9 in healthy animals. Therefore, higher serum concentrations were maintained for longer periods of time in animals with ALI compared to healthy animals. Similar effects of drug lung retention and prolongation of the clearance of temafloxacin and sparfloxacin were described in a mice infected with *Streptococcus pneumoniae* [35].

One limitation of this study is the challenge of drug administration by nebulisation in small animal models, which is challenging due to multiple factors. The administration of the drug by nebulisation to the animals is challenging due to multiple factors. Contrary to humans, only spontaneous respiratory activity can be studied, and animals with ongoing ALI are expected to have altered breathing patterns compared to healthy animals. Hyperventilation with smaller volumes can be expected, which may alter the deposition of inhaled aerosol in different parts of the respiratory system compared with healthy individuals. However, analogical changes in breathing patterns can be expected in patients with influenza infection.

We determined zanamivir bioavailability following nebulisation of 1.9%. This is in line with the data from clinical trials that described the bioavailability of zanamivir by other types of INH administration, such as dry powder inhalation, intranasal drops, and intranasal spray of 13.2%, 2.3%, and 1.6%, respectively [5,36].

A 600 mg IV dose of zanamivir in healthy humans achieved lung lining fluid concentrations comparable to a 10 mg INH dose using DPI [37]. However, if there was a similar change in zanamivir permeability in patients with influenza pneumonia as we observed in our study with LPS-induced ALI, then an IV dose of 600 mg might not have corresponded to 10 mg INH as it did in healthy volunteers and sufficient target concentrations in lungs may not have been achieved.

This study demonstrates the PK superiority of INH administration to achieve local intrapulmonary exposition and indicate that ALI significantly impairs zanamivir penetration into the lungs from systemic circulation. Further investigation is required to better understand the mechanism underlying these changes. Specifically, it remains unclear how much of the drug is taken up by lung immune cells, particularly alveolar macrophages. Future studies should aim to quantify drug concentrations within macrophages and assess whether inflammation alters intracellular drug accumulation.

Moreover, it would be valuable to compare the pulmonary distribution of other antimicrobial agents. Determining whether the observed reduction in lung penetration is specific to BCS IV drugs or more precisely zanamivir, or represents a broader limitation applicable to other drugs under inflammatory conditions. Such insights could inform the rational selection of compounds and the development of optimized formulations for respiratory infections.

The findings of this study may have consequences for zanamivir drug delivery into the site of action in patients with influenza and the efficacy of the treatment. However, confirmation in clinical trials is necessary, as data from animal models may not be directly translatable to human disease. Nonetheless, these results highlight important changes in drug PK and lung penetration under inflammatory conditions. They provide a strong basis for further clinical research, particularly in patients with acute or chronic lung inflammation, where similar pathophysiological alterations may affect drug distribution and therapeutic outcomes.

## Supplementary Material

Supplementary figure 3.tif

Supplementary figure 1.tif

Supplementary legends R1 clean.docx

Supplementary figure 2.tif

## Data Availability

All data generated or analysed during this study were included in the published article. Raw data that support the findings of this study are openly available in OSF repository at https://osf.io/jfw7q/?view_only=cebbecc0b06a4bacb909bba21f2e8b38. Further inquiries about the dataset can be directed to the corresponding author on reasonable request.
