# Supplementary figures and images for: Zanamivir exposure in healthy rats and rats with acute lung injury
Source: Ann Med. 2025 Jul 20;57(1):2534523. doi: 10.1080/07853890.2025.2534523 (PMC12278471; doi:10.1080/07853890.2025.2534523)

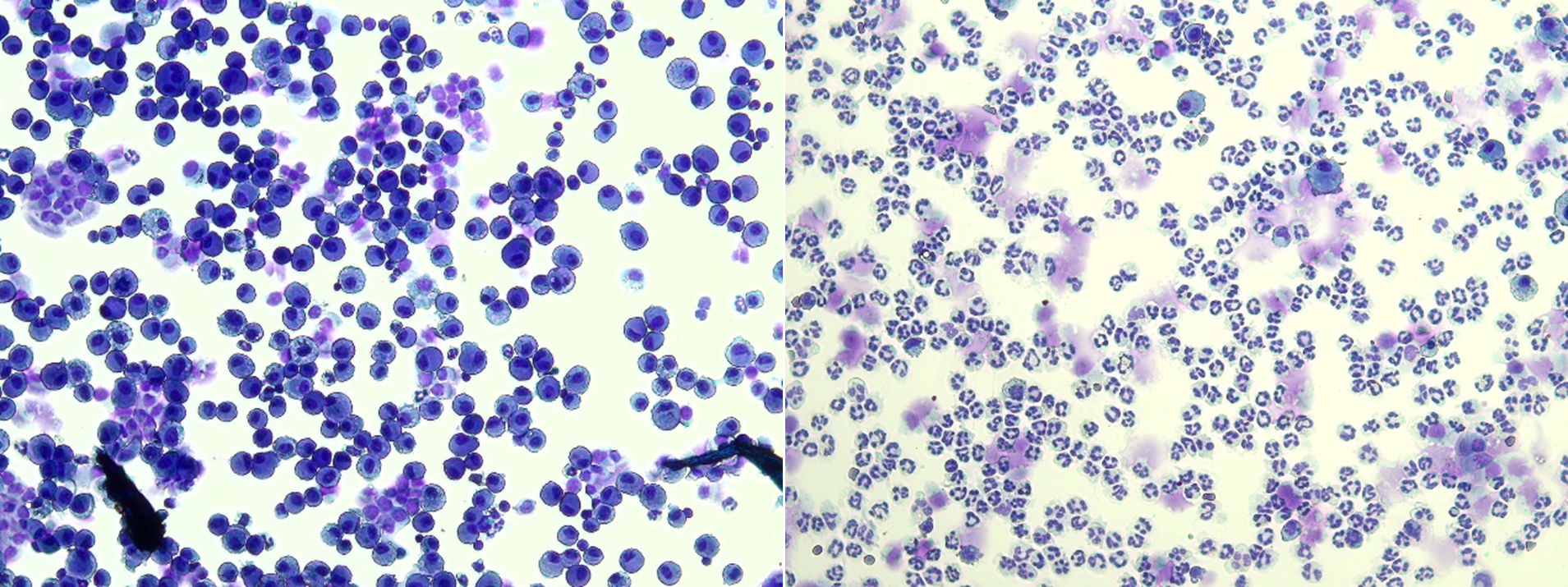

Supplement: Supplementary figure 3.tif [file IANN_A_2534523_SM2325.tif]

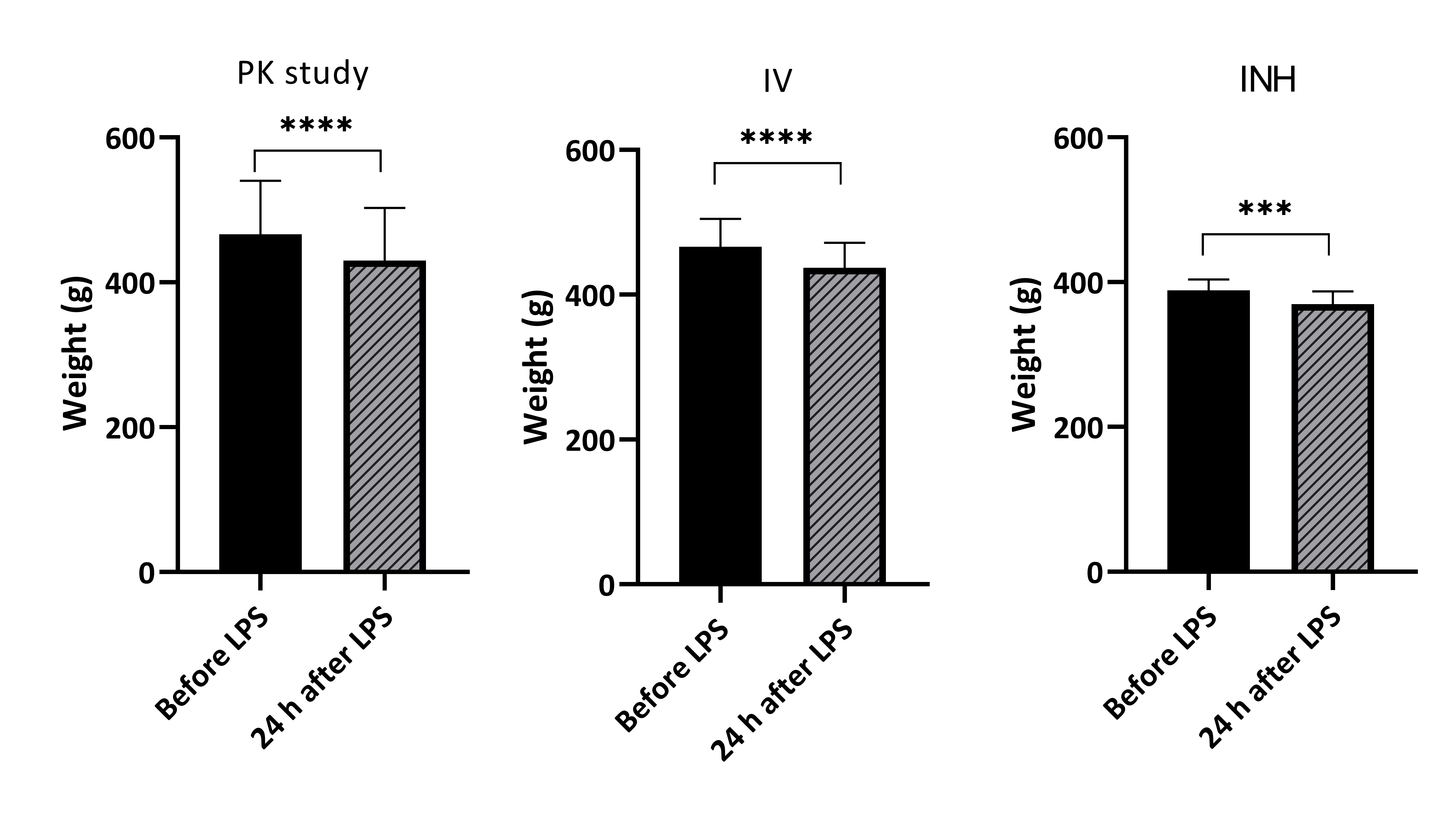

Supplement: Supplementary figure 1.tif [file IANN_A_2534523_SM2324.tif]

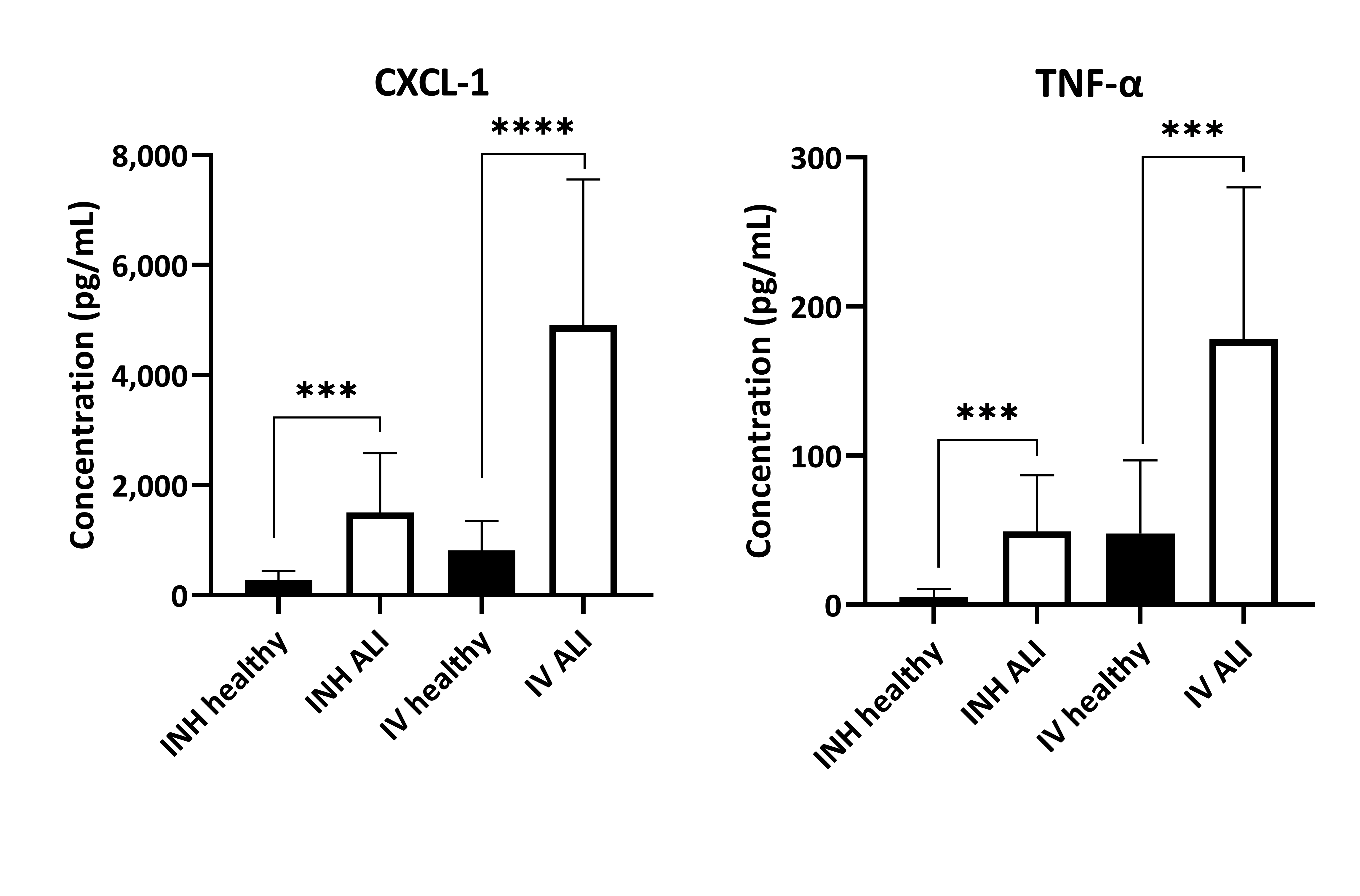

Supplement: Supplementary figure 2.tif [file IANN_A_2534523_SM2322.tif]
