# Supplementary material for: Zanamivir exposure in healthy rats and rats with acute lung injury
Source: Ann Med. 2025 Jul 20;57(1):2534523. doi: 10.1080/07853890.2025.2534523 (PMC12278471; doi:10.1080/07853890.2025.2534523)
Supplement: Supplementary legends R1 clean.docx [file IANN_A_2534523_SM2323.docx]

**Supplementary data**

Supplementary figure 1: Comparison of body weights (mean ± SD) before and 24 hours after lipopolysaccharides (LPS) administration in three study groups: pharmacokinetic (PK) study, intravenous (IV) and inhalation (INH) drug administration in bronchoalveolar lavage penetration studies. ***(P≤0.001). ***(P≤0.0001).

*Supplementary figure 2*: Concentration of TNF-α and CXCL-1 in bronchoalveolar lavage samples in healthy rats (solid bars) and rats with induced acute-lung injury (open bars) after intravenous (IV) and inhalation (INH) drug administration. The values are represented as mean ± SD concentrations. *** (P≤0.001). **** (P≤0.0001).

*Supplementary figure 3*: Cells found in bronchoalveolar lavage samples obtained from healthy rat (left) and rat with induced acute-lung injury (right). Modified by May-Grunwald-Giemsa staining.
